# Knowledge, attitude, and uptake of human papillomavirus vaccine among adolescent schoolgirls in Ethiopia: a systematic review and meta-analysis

**DOI:** 10.1186/s12905-023-02412-1

**Published:** 2023-05-20

**Authors:** Dagne Addisu, Natnael Atnafu Gebeyehu, Yismaw Yimam Belachew

**Affiliations:** 1 Department of Midwifery, College of Health Sciences, Debre Tabor University, Debre Tabor, Ethiopia; 2 School of Midwifery, College of Health Science and Medicine, Wolaita Sodo University, Wolaita Sodo, Ethiopia; 3 Department of Obstetrics and Gynecology, School of Medicine, College of Health Sciences, Debre Tabor University, Debre Tabor, Ethiopia

**Keywords:** Knowledge, Attitude, Uptake, HPV vaccine, Adolescents, Ethiopia

## Abstract

**Background:**

Cervical cancer is an international public health issue. Nearly all cases of cervical cancer are caused by the human papillomavirus. The HPV vaccine prevents more than 75% of cervical cancer. The extent to which adolescent girls' knowledge and uptake of the HPV vaccine have to be investigated in order to build effective promotion strategies and increase the uptake of the vaccine. The evidence that is currently available in this area is controversial and inconclusive. Hence, this study has estimated the pooled proportion of good knowledge, positive attitude, and uptake of the HPV vaccine and its associated factors among adolescent schoolgirls in Ethiopia.

**Methods:**

PubMed, Google Scholar, AJOL, ScienceDirect, and DOAJ were used to search relevant studies. A total of 10 studies were included. The data were extracted by two reviewers using Microsoft Excel and exported to STATA Version 17 for analysis. A random effects model was applied during the analysis. Heterogeneity and publication bias across the studies were evaluated using I^2^ statistics and Egger’s test, respectively. The PROSPERO registration number for the review is CRD42023414030.

**Result:**

A total of eight studies comprising 3936 study participants for knowledge and attitude and five studies with 2,481 study participants for uptake of HPV were used to estimate the pooled proportions of good knowledge, a positive attitude, and uptake of the HPV vaccine, respectively. The pooled proportions of good knowledge, positive attitude, and uptake of the HPV vaccine were 55.12%, 45.34%, and 42.05%, respectively. Being an urban resident (OR = 4.17, 95% CI = 1.81, 9.58), having good knowledge (OR = 6.70, 95% CI = 3.43, 13.07), and a positive attitude (OR = 2.04, 95% CI = 1.51, 2.74), were significantly associated with the uptake of the vaccine.

**Conclusion:**

The pooled proportions of good knowledge, a positive attitude, and uptake of the HPV vaccine were low in Ethiopia. Being an urban resident and having good knowledge and a positive attitude towards the HPV vaccine were significantly associated with the uptake of the HPV vaccine. We recommend increasing adolescent knowledge, positive attitudes, and uptake of HPV vaccination through school-based seminars, health education, and community mobilization.

**Supplementary Information:**

The online version contains supplementary material available at 10.1186/s12905-023-02412-1.

## Background

Cervical cancer is an international public health issue [1]. Globally, it is the 4th most common cancer among women [2–4]. Worldwide, around 604,127 new cervical cancer cases and 341,831 deaths occurred in 2020 [2]. The majority of cases (90%) and 85% of these deaths occur in low and middle-income countries, with Sub-Saharan Africa countries having the highest rate of cervical cancer mortality [5, 6]. In Ethiopia, cervical cancer is the 2nd most common female cancer, with an estimated 7,445 newly diagnosed cases in 2020 [2].

Nearly all cases of cervical cancer and over 70% of malignancies affecting the vagina, vulva, and oropharynx are caused by the human papillomavirus (HPV) [7]. HPV is the most prevalent viral infection of the reproductive tract, with most women experiencing it shortly after becoming sexually active [8]. There are more than 100 distinct HPV types, and around 30 of them affect the genitalia [9]. High-risk or oncogenic types of HPV, mainly HPV types 16 and 18, cause 70% of all cases of cervical cancer [9–11].

The HPV vaccine is the most important preventive measure for HPV-associated cancers [12] and prevents more than 75% of occurrences of cervical cancer [13]. The World Health Organization has recommended the introduction of HPV vaccines into children's and adolescents' immunization programs [14]. Despite this, only 1.4% of all eligible girls have received a full course of HPV vaccination, leaving the HPV vaccine with disappointingly low global coverage [6, 15]. Evidence found that the full course vaccine coverage among adolescent girls (10–20 years) was 1.2% in Africa, 1.1% in Asia, 31.1% in Europe, 19% in Latin America and the Caribbean, 35.6% in North America, and 35.9% in Oceania in 2014 [15].

The bivalent HPV vaccine was introduced in Ethiopia on December 3rd, 2018 for girls who are 14 years of age [16]. The vaccine is delivered mainly in health facilities and through a school-based approach to reach all 14-year-old girls [16]. However, the coverage of the HPV vaccine is low in Ethiopia and ranges from 15% [17] to 66.5% [18].

Even though the HPV vaccine is proven to reduce cervical cancer incidence, many factors influence HPV vaccine uptake. The rate of HPV vaccine uptake has been shown to vary by knowledge and attitude towards HPV vaccine, in addition to other factors such as adolescents' age, social influence, lack of health education on HPV vaccine, and parents' educational status [19–21]. Understanding the diverse multilevel factors associated with HPV vaccine initiation and completion is the most important strategy to improve HPV vaccine coverage [9].

Though many efforts have been made to study knowledge, attitude, and uptake of the HPV vaccine, primary studies reporting the proportion of good knowledge, a positive attitude, and uptake of the vaccine are fragmented. The evidence that is currently available in this area is controversial and inconclusive. Therefore, this meta-analysis aimed to estimate the pooled proportion of good knowledge, a positive attitude, and uptake of the HPV vaccine and its associated factors in Ethiopia. The result of this study will provide important input for policymakers to improve the uptake of the HPV vaccine by creating a positive attitude and good knowledge about the vaccine.

## Methods

This systematic review and meta-analysis aimed to estimate the pooled proportion of good knowledge, positive attitude, and uptake of the human papillomavirus vaccine and associated factors among adolescent schoolgirls in Ethiopia. The protocol of this meta-analysis was registered at  PROSPERO with a registration number of CRD42023414030. A standard PRISMA checklist was used to present the findings (S1 Table).

### Databases and search strategies

All relevant studies were systematically searched in the following electronic databases: PubMed/MEDLINE, Google Scholar, African Journal of Online (AJOL), ScienceDirect, and DOAJ). Initially, studies were exhaustively searched by using the full title (“knowledge, attitude, and uptake of HPV vaccine and associated factors among adolescent schoolgirls in Ethiopia”) and keywords ("knowledge", "attitude", "uptake", "human papillomavirus", "vaccine", "associated factors", "adolescent", "schoolgirls", "Ethiopia"). The Boolean operators "OR" or "AND" were used in conjunction with or independently of one another to connect these keywords to form search terms. In addition, reference lists for all included research were reviewed to identify any missed studies. Furthermore, institutional repositories of Ethiopian universities, primarily those in Gondar, Jimma, Addis Abeba, and Harameya University, were examined for unpublished papers. Searching details for PubMed were as follows: ("knowledge"[MeSH Terms] OR "knowledge"[All Fields]) AND ("attitude"[MeSH Terms] OR "attitude"[All Fields]) AND ("uptake" [All Fields] OR "Practice"[All Fields] OR "utilization" [All Fields]) AND ("papillomavirus vaccines"[MeSH Terms] OR ("papillomavirus"[All Fields] AND "vaccines"[All Fields]) OR "papillomavirus vaccines"[All Fields] OR ("human"[All Fields] AND "papilloma"[All Fields] AND "virus"[All Fields] AND "vaccine"[All Fields]) OR "human papilloma virus vaccine"[All Fields]) AND associated[All Fields] AND factors[All Fields] AND ("adolescent"[MeSH Terms] OR "adolescent"[All Fields]) AND ("schoolgirls" [All Fields] OR "females" [All Fields] OR "young adults"[All Fields]) AND ("Ethiopia"[MeSH Terms] OR "Ethiopia"[All Fields]). The search period was restricted between January 1, 2015 and December 23, 2022 (S2 Table).

### Eligibility criteria

We specified eligibility criteria for the search and meta-analyses using the condition, context, and population (CoCoPop) framework.

#### Inclusion criteria

This systematic review and meta-analysis included studies that fulfilled the following criteria:

- **Condition (Co):** We included studies that examined at least one or more of the following key outcomes: 1) the proportion or level of good knowledge; 2) the proportion or level of a positive attitude; 3) studies that report HPV vaccination coverage; 4) studies that examined factors associated with HPV vaccine uptake among adolescent schoolgirls.
- **Context (Co):** We included studies that were conducted in Ethiopia.
- **Population (Pop):** Studies that were done among female adolescents (9–14 years)
- **Study Design**: We included cross-sectional and/or case–control studies.
- **Publication status:** Both published and unpublished (pre-print) studies
- **Language:** We included all studies written in the English language.

##### Exclusion criteria

Qualitative studies and studies with a different study population as well as different outcomes in terms of interests were excluded.

### Outcome measurement

The primary outcome was HPV vaccine knowledge, attitude, and uptake rate among adolescent schoolgirls. The secondary outcomes were factors associated with HPV vaccine uptakes such as background characteristics, HPV vaccine attitudes, HPV knowledge, and HPV vaccine availability.

#### Uptake

Uptake of the HPV vaccine was defined as the proportion of those adolescent schoolgirls who had received at least one dose of the HPV vaccination.

#### Knowledge

Knowledge of adolescent schoolgirls towards HPV vaccine was measured by asking 7 knowledge-related questions and a score greater than the mean was classified as good knowledge [18, 22–26].

#### Attitude

Attitude towards HPV vaccination was measured by using seven attitude-related questions and participants with scores above the mean were classified as having a ‘positive attitude’ towards HPV vaccination [18, 22–27].

### Study selection and quality assessment

All studies retrieved through the search were exported to Endnote version 7 (Thomson Reuters, USA) software. After duplicates were removed, two authors (DA and NG) independently assessed titles and abstracts for eligibility criteria using Endnote software and manually. During the screening, each study that did not fulfill the inclusion criteria was excluded, and the reasons for the exclusion were documented. A consensus approach with input from the third reviewer (YB) was applied to resolve disagreements. Finally, a modified Newcastle–Ottawa quality assessment tool adapted for cross-sectional studies was used to assess the quality of the studies that fulfilled the inclusion criteria [28]. The quality of the studies was assessed by two authors (DA and NG) individually. The quality assessment tool has three major themes and a total of 10 scores or points: selection (maximum 5 points), comparability (maximum 2 points), and outcomes (3 points). Finally, articles that had a score of ≥ 7 points out of 10 were considered high-quality and included in this study (S3 Table).

### Data extraction process

A predesigned data extraction form was used to extract all necessary data. The data extraction form was prepared in Microsoft Excel and contains the first author's name, publication year, study region, study setting, study period, sample size, the proportion of good knowledge, positive attitude, and uptake of HPV vaccine, as well as an adjusted odds ratio (AOR) with a 95% confidence interval for significant risk factors for the uptake of HPV vaccine (S4 Table). The data were extracted by two reviewers (DA and YB).

### Data synthesis and statistical analysis

A random effects model was used to determine the pooled proportion of good knowledge, positive attitude, and uptake of the HPV vaccine and associated factors among adolescent schoolgirls in Ethiopia [28–30]. Subgroup analysis and sensitivity analysis were performed to identify the source of heterogeneity. Finally, Egger’s regression test was done to assess publication bias [31].

## Result

### Search result

**Fig. 1:**
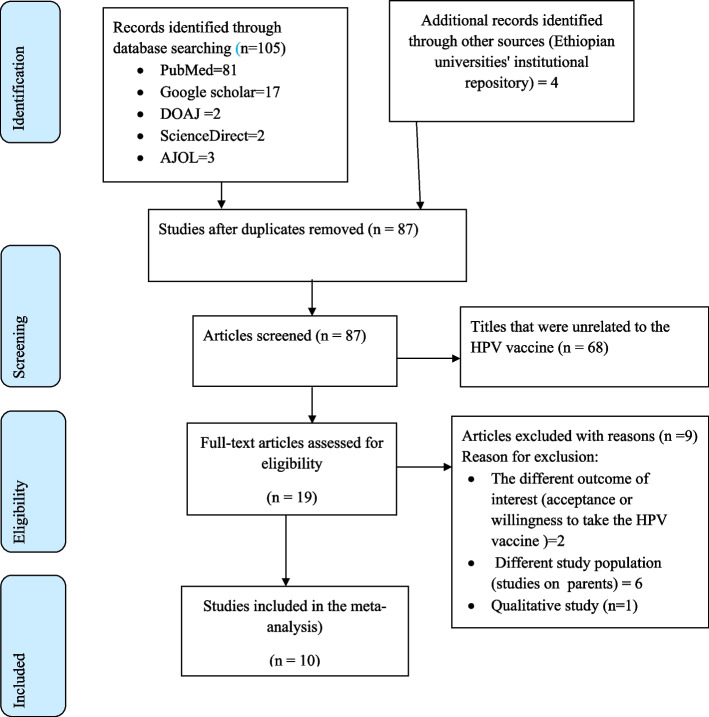
Schematic presentation of study selection for systematic review and meta-analysis of knowledge, attitude, and uptake of HPV vaccine and associated factors among adolescent schoolgirls in Ethiopia

A total of 109 studies were identified from different international databases and institutional repositories at Ethiopian universities. The Endnote 7 reference manager was used to screen retrieved studies. Then, a total of 99 articles were removed because of unrelated papers to the HPV vaccine, duplicate findings, different study populations, and different outcomes of interest. Ten studies that met the inclusion criteria were finally included in this analysis (Fig. 1).

### Characteristics of included studies

A total of 10 studies were included in this systematic review and meta-analysis [17, 18, 22–27, 32, 33]. Among 10 studies, 8 studies were used to estimate the pooled proportion of both good knowledge and positive attitude about the HPV vaccine; while 5 studies were used to determine the pooled uptake of the HPV vaccine. All the included studies used a cross-sectional study design. A total of 4,794 study participants were involved to estimate the pooled level of good knowledge, positive attitude, and uptake of the HPV vaccine. Three regions were represented in this study: four studies were from the Oromia region, three studies were from the South Nation Nationalities and Peoples Region (SNNPR), and three studies were from the Amhara region (Table 1).

**Table 1:** Characteristics of included studies to estimate the pooled proportion of good knowledge, positive attitude, and uptake of HPV vaccine in Ethiopia

Authors	Publication year	Study area	Region	Sample size	Good knowledge (%)	Positive attitude (%)	Uptake of HPV vaccine (%)	Data collection period
Geneti et al	2016	Jimma	Oromia	448	5.80	44.4	N/A	February 10 -16, 2016
Beyen et al	2021	Ambo	Oromia	422	88.6	55.6	44.4	December 1–30, 2020
Ukumo et al	2021	Arba Minch	SNNPR	516	71.7	51	N/A	January 1, 2020
Tesfaye et al	2017	Gondar	Amhara	267	N/A	N/A	15	April 1 to May 30, 2016
Kassa et al	2021	Minjar-Shenkora	Amhara	591	N/A	N/A	66.5	February 1–30, 2020
Biyazin et al	2022	Jimma	Oromia	366	52.7	31.4	N/A	January 25 to 29, 2021
Lakneh et al	2022	Bahir Dar	Amhara	633	58.1	16	45.3	March 1–30, 2021
Bulto et al	2021	Ambo	Oromia	422	24.9	55.6	N/A	January 20–26, 2020
Ukumo et al	2022	Arba Minch	SNNPR	561	75.2	51	N/A	January 1, 2020
Terefe et al	2022	Wolaita Sodo	SNNPR	568	64	58	42	February 10–15, 2021

*N/A* Not applicable (the study did not assess that specific outcome)

### Pooled estimates of adolescent schoolgirls’ knowledge, attitude, and uptake of the human HPV vaccine

**Fig. 2:**
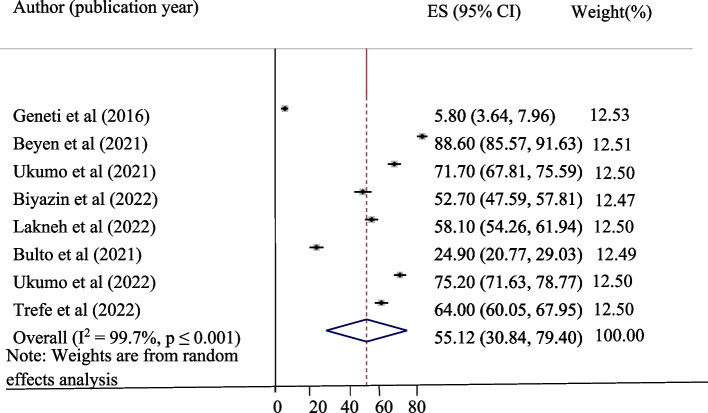
Pooled level of good knowledge about HPV vaccine among adolescent schoolgirls

**Fig. 3:**
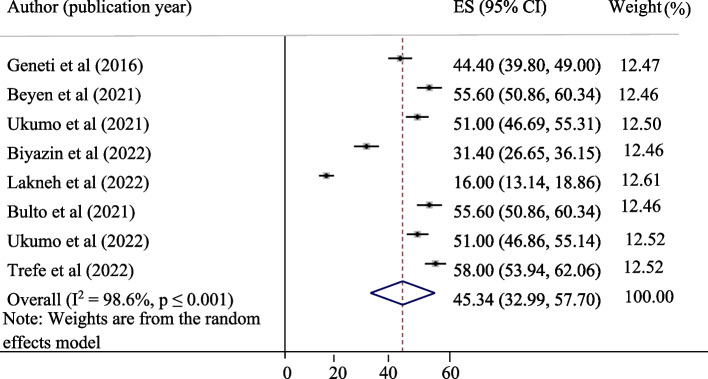
Pooled level of positive attitude about HPV vaccine among adolescent schoolgirls in Ethiopia

**Fig. 4:**
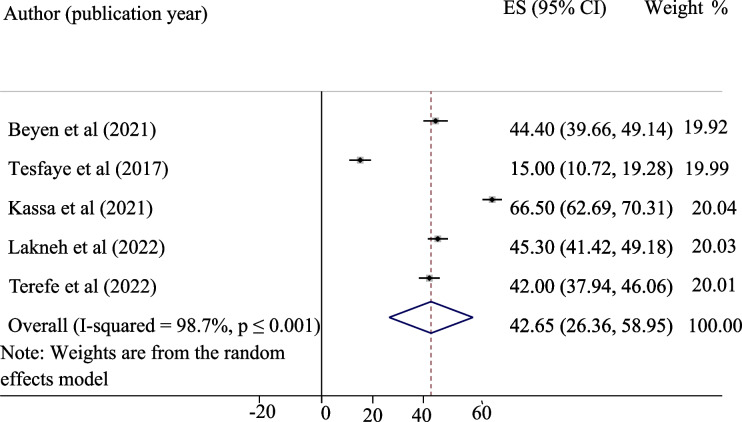
Pooled coverage of HPV vaccine uptake among adolescent schoolgirls in Ethiopia

The pooled level of good knowledge, positive attitude, and uptake of the HPV vaccine in Ethiopia was presented in a forest plot. The pooled level of good knowledge, positive attitude, and uptake of the HPV vaccine in Ethiopia was 55.12%, 45.34%, and 42.05%, respectively. High heterogeneity was observed across the studies, with an I^2^ value of 99.7%, 98.6%, and 98.7% for good knowledge, positive attitude, and uptake of the HPV vaccine, respectively (Figs. 2, 3 and 4).

### Subgroup analysis

Subgroup analysis was done to identify the source of heterogeneity and minimize the random variations between the point estimates of the primary studies and the pooled estimate by using geographical regions of the country and study settings. However, we did not find a possible reason for this high heterogeneity. The pooled estimate of good knowledge was highest in studies conducted at the South Nation Nationalities and Peoples Region (SNNPR) and primary schools, with pooled proportions of 70.34% and 78.55%, respectively. Regarding attitude, the pooled proportion of positive attitude was highest in studies conducted at SNNPR and primary schools, with proportions of 53.37% and 52.36%, respectively. Concerning the uptake of the HPV vaccine, the pooled uptake of the vaccine was found to be 42.28% in studies conducted in the Amhara region and 55.50% in studies conducted at primary schools (Table 2).

**Table 2:** Subgroup analysis of good knowledge and positive attitude about HPV vaccine and its uptake among adolescent students in Ethiopia

**Knowledge related articles**			
Variable	Characteristics	**Pooled levels of good knowledge** (95%CI)	I^2^ and P-value
Study setting	University	34.88 (22.16, 91.91)	(99.8, p ≤ 0.001)
	Secondary school (grade 9–12)	45.22 (24.00, 66.45)	(98.6%, p ≤ 0.001)
	Primary school (grade 7–8)	78.55 (67.98, 89.13)	(96.4%, p ≤ 0.001)
Study region	Amhara	58.10(54.26, 61.94)	NR
	Oromia	42.99(0.35, 85.64)	(99.8%, p ≤ 0.001)
	SNNPR	70.34(63.87, 76.81	(88.5%, p ≤ 0.001)
**Attitude related articles**			
Variable	Characteristics	**Pooled proportion of positive attitude** (95%CI)	I^2^ and P-value
Study setting	University	51.24 (37.92, 64.57)	(94.7%, p ≤ 0.001)
	Secondary school (grade 9–12)	34.28 (10.86, 57.70)	(99%, p ≤ 0.001)
	Primary school (grade 7–8)	52.36 (49.48, 55.24)	(22.8, p = 0.274)
Region	Amhara	16(13.14, 18.86)	NR
	Oromia	46.75(35.56, 57.94)	(95.6%, p ≤ 0.001)
	SNNPR	53.37(48.73, 58.00)	(73%, p = 0.025)
**HPV vaccine uptake-related articles**			
Variable	Characteristics	**Pooled levels of HPV vaccine uptake** (95%CI)	I^2^ and P-value
Study setting	University	28.51(2.05, 54.97)	(98.8%, p ≤ 0.001)
	Secondary school (grade 9–12)	45.3(41.42, 49.18)	NR
	Primary school (grade 7–8)	55.50 (33.84, 77.15)	(98%, p ≤ 0.001)
Region	Amhara	42.28(13.62, 70.95)	(99.4%, p ≤ 0.001)
	Oromia	44.40(39.66, 49.14)	NR
	SNNPR	42(37.94, 46.06)	NR

*NR* Not reported. Since there was a single study in that specific category, I^2^ and P-values were not calculated

### Sensitivity analysis

A sensitivity analysis was done to check the influences of individual studies on the pooled estimate of good knowledge, a positive attitude, and uptake of the HPV vaccine. The result showed that there was no significant influence of individual study on the pooled effects of good knowledge, a positive attitude, and the uptake of the HPV vaccine (Table 3).

**Table 3:** Results of sensitivity analysis for good knowledge, positive attitude, and uptake of human papillomavirus vaccine among adolescent schoolgirls in Ethiopia

**Knowledge related articles**		
**Study omitted**	**Pooled Estimate**	**[95% Conf. Interval]**
Geneti et al. (2016) [32]	62.20	46.85, 77.55
Beyen et al. (2021) [23]	50.32	26.30, 74.35
Ukumo et al. (2021) [25]	52.75	25.87, 79.63
Biyazin et al. (2022) [26]	55.46	28.51, 82.41
Lakneh et al. (2022) [27]	54.69	27.08, 82.30
Bulto et al. (2021) [33]	59.43	32.54, 86.32
Ukumo et al. (2022) [24]	52.25	25.56, 78.93
Terefe et al. (2022) [22]	53.85	26.55, 81.15
**Combined**	**55.12**	**30.83, 79.40**
**Attitude related articles**		
**Study omitted**	**Pooled Estimate**	**[95% Conf. Interval]**
Geneti et al. (2016) [32]	45.48	31.41, 59.55
Beyen et al. (2021) [23]	43.88	30.40, 57.36
Ukumo et al. (2021) [25]	44.54	30.64, 58.43
Biyazin et al. (2022) [26]	47.33	33.55, 61.11
Lakneh et al. (2022) [27]	49.59	43.05, 56.14
Bulto et al. (2021) [33]	43.88	30.40, 57.36
Ukumo et al. (2022) [24]	44.53	30.59, 58.48
Terefe et al. (2022) [22]	43.53	30.31, 56.75
**Combined**	**45.34**	**32.99, 57.69**
**Uptake of HPV vaccine**		
**Study omitted**	**Pooled Estimate**	**[95% Conf., Interval]**
Beyen et al. (2021) [23]	42.21	21.83, 62.59
Tesfaye et al. (2017) [17]	49.57	37.87, 61.27
Kassa et al. (2021) [18]	36.67	22.56, 50.78
Lakneh et al. (2022) [27]	41.98	20.60, 63.36
Terefe et al. (2022) [22]	42.81	21.69, 63.92
**Combined**	**42.65**	**26.35, 58.94**

### Publication bias

Publication biases were assessed objectively by using Egger's regression test, and the results of Egger's regression test indicated that there was no publication bias or small study effects between the studies with a *p*-value of 0.150 for good knowledge, 0.20 for a positive attitude, and 0.408 for uptake of the HPV vaccine.

### Factors associated with the uptake of the HPV vaccine

#### Being an urban resident and uptake of the HPV vaccine

**Fig. 5:**
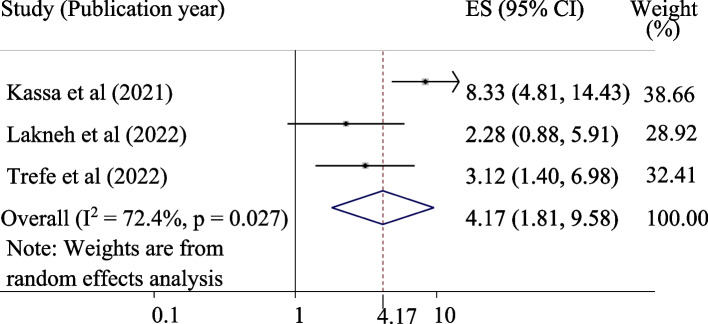
Forest plot for the association of urban resident with the uptake of HPV vaccine among adolescent schoolgirls in Ethiopia

The association between urban resident and uptake of the HPV vaccine was evaluated by three studies [18, 22, 27]. The pooled effect of being an urban resident was significantly associated with the uptake of the HPV vaccine. Adolescent schoolgirls from urban areas were 4.17 times more likely than those from rural areas to receive the HPV vaccine (odds ratio = 4.17, 95% CI = 1.81, 9.58) (Fig. 5).

#### Positive attitudes about the HPV vaccine and its effect on the uptake

**Fig. 6:**
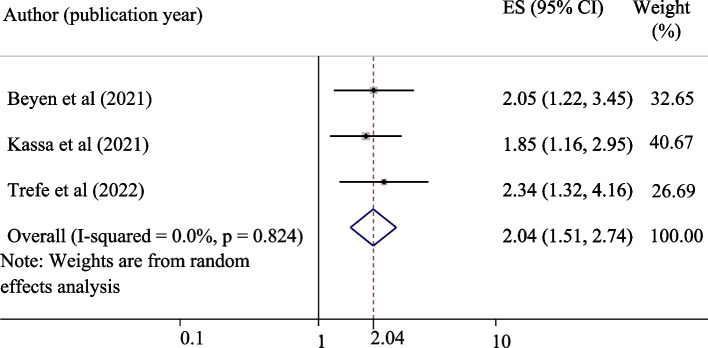
Forest plot for the association of positive attitude about HPV vaccine with the uptake of the vaccine among adolescent schoolgirls in Ethiopia

Three studies were used to assess the pooled effects of positive attitudes about the HPV vaccine on the uptake of the vaccine [18, 22, 23]. The result revealed that having a positive attitude about the HPV vaccine was significantly associated with the uptake of the vaccine. Those adolescent schoolgirls who had a positive attitude about the HPV vaccine were 2.04 times more likely to uptake the vaccine as compared to those who had a negative attitude (OR = 2.04, 95% CI = 1.51, 2.74) (Fig. 6).

#### The association between good knowledge about HPV vaccine and vaccine uptake

**Fig. 7:**
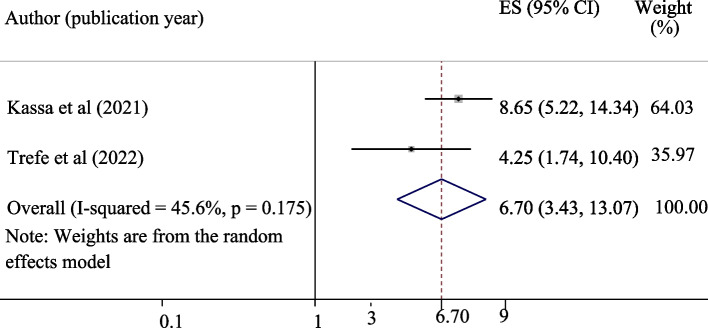
Forest plot for the association of good knowledge about HPV with the uptake of the vaccine among adolescent schoolgirls in Ethiopia

The effect of good knowledge on the uptake of the HPV vaccine was evaluated by using two studies [18, 22]. The pooled effect of having good knowledge was significantly associated with the uptake of the HPV vaccine (OR = 6.70, 95% CI = 3.43, 13.07). Those adolescent schoolgirls who had good knowledge were 6.7 times more likely to take the HPV vaccine as compared to those who had poor knowledge (Fig. 7).

## Discussion

This systematic review and meta-analysis has estimated the pooled proportion of good knowledge, positive attitude, and uptake of the HPV vaccine and its associated factors among adolescent schoolgirls in Ethiopia. To the best of our knowledge, this meta-analysis is the first of its kind in determining the pooled proportion of good knowledge, a positive attitude, and uptake of the HPV vaccine and these findings may enable policymakers to design strategies for the improvement of knowledge, attitude, and uptake of the HPV vaccine among adolescents.

The pooled proportion of good knowledge about the HPV vaccine was found to be 55.12%, with a 95% CI of 30.84 to 79.40. This finding was in line with a systematic review and meta-analysis finding in European countries, which have 51.8% of good knowledge [34]. However, this finding was higher than a meta-analysis study in China, which has 17.55% of good knowledge [35]. The variation in the level of knowledge could be attributed to differences in governmental and public concerns about cervical cancer and the HPV virus across countries. Cervical cancer is the second-leading gynecology cancer in Ethiopia, and the federal minister of health has placed special emphasis on the primary prevention of this cancer by providing the HPV vaccine [36]. This high level of governmental and public concern about cervical cancer and its prevention strategies may lead to a relatively higher proportion of HPV vaccine knowledge as compared to the previous study in China. In addition, variations in study periods might contribute to this difference. In this regard, the majority of the studies included in this analysis were done more recently as compared to the previous study in China, and there have been high concerns and attention about HPV and cervical cancer in recent years that might lead to better knowledge about the HPV vaccine.

Regarding attitude, the pooled estimate of positive attitude towards the HPV vaccine was found to be 45.34% with a 95% CI of 32.99–57.70. This finding was consistent with research finding in the United States, which has 51% of positive attitude [37]. This outcome, meanwhile, was less favorable than the results of countrywide research in Hungary, where 80% of respondents indicated they had a favorable attitude about receiving the vaccination [38]. This might be due to differences in the burden of cervical cancer and HPV infection between the two countries. Among the European Union's member states, Hungary ranks fourth in terms of prevalence and fifth in terms of the mortality of cervical cancer. This high burden of cervical disease might enable adolescents to have a better attitude about the HPV vaccine as compared to our country [38]. Moreover, this finding was lower than a population-based study in Germany, which had 61.5% positive attitudes [19]. This higher level of positive attitude in Germany might be due to the large sample size (4,747) as compared to our study (3936 study participants).

In this study, the overall pooled proportion of uptake of the HPV vaccine was 42.05%, with a 95% CI of 26.36 to 58.95. This finding was consistent with National Health Survey (NHS) report in Brazilian [7] and Tanzania [39], with 58.4% and 49% coverage of the HPV vaccine, respectively. This finding was also in line with a meta-analysis study in high and low-income countries, which was 41.5% [40]. However, the current result was higher than the finding of a meta-analysis study in a less developed region, which has a 2.7% of HPV vaccine coverage [15]. This discrepancy might be due to variations in vaccine delivery strategies. In Ethiopia, the vaccine is delivered through a school-based approach, which may cover a significant number of targeted groups (adolescents). However, some countries included in the previous meta-analysis did not use a school-based vaccine delivery approach [15]. School-based vaccination strategy is considered the most effective and efficient means of ensuring high vaccine coverage for adolescents [41]. Another evidence also revealed that counties using school-based delivery strategies for HPV vaccination have a higher uptake as compared to out-of-school approaches or health facility vaccine delivery strategies [42]. The higher uptake rates in this study imply that a school-based strategy can remove some obstacles and raise the possibility that adolescents will enroll in the program.

The uptake of the HPV vaccine among adolescent schoolgirls in Ethiopia was also higher than in the Demographic and Health Survey report in Uganda. A multilevel analysis from the 2016 Demographic and Health Survey data in Uganda indicated that only 22% of girls aged 10–14 years uptake the HPV vaccine [43]. The variation between the two countries might be due to differences in cultural and social issue and the level of attitude about HPV vaccine. This low HPV vaccine coverage in Uganda may be attributed to the presence of unfavorable attitudes towards the vaccine [44] and technical difficulties with the school-based HPV vaccine delivery method [43, 45].

On the other hand, the uptake of HPV vaccine was lower than a study finding in the USA, which has 62.8% HPV vaccine coverage [46]. This could be due to differences in the accessibility or availability of the HPV vaccine. In Ethiopia, the availability of the HPV vaccine was relatively low compared to the USA. Even though there is a high level of willingness or acceptance to take the HPV vaccine among adolescents in Ethiopia, the vaccine is not available everywhere [47, 48]. So, low accessibility or availability may be the possible reason for low HPV vaccine coverage in Ethiopia as compared to the previous study in the USA. Moreover, the uptake of the HPV vaccine among adolescent schoolgirls in Ethiopia was also lower than a natation wide study in Australian. The national HPV vaccination coverage for girls aged 12–17 years in Australia was 83% for dose 1, 78% for dose 2, and 70% for dose 3 [49]. The low HPV vaccine coverage in our country might be due to a variety of reasons, such as poor perception, fear of side effects, a low level of awareness, poor knowledge about the HPV vaccine, a negative attitude, and misunderstandings about the HPV vaccine [23, 27].

This study found that having good knowledge about the HPV vaccine was significantly associated with the uptake of the HPV vaccine. Evidence from a meta-analysis study supports the current finding [50]. This finding was also supported by studies done in Italy [51] and China [52]. The relationship could be explained by the idea that knowledge is the key to implementing preventative measures against specific diseases and improving the health status of an individual. This finding reflects the need for improving adolescents' knowledge about HPV infections and its preventive strategies including the significance of vaccination through different strategies.

Furthermore, this study also found that having a positive attitude about the HPV vaccine was significantly associated with the uptake of the HPV vaccine. A systematic review and meta-analysis study supports the current finding [50]. Other evidence from Uganda also supported the present finding [53]. This may be explained by the fact that those adolescents' motivation and initiatives to do something or maintain a healthy lifestyle are significantly impacted by their attitudes, where favorable views enhance people's performance and the application of preventive measures.

Lastly, being an urban resident was significantly associated with the uptake of the HPV vaccine. This could be justified because adolescent schoolgirls from urban areas may be more aware of the vaccine's benefits, which may have influenced them to take the vaccine. This finding suggests the need for improving health promotion in rural settings and providing access to the HPV vaccine for all adolescents in rural areas.

This study should be interpreted in light of the following limitations: The lack of studies from some regions might affect the generalization. In addition, there was heterogeneity across studies, which might affect the pooled estimate of good knowledge, a positive attitude, and the uptake of the human papillomavirus vaccine. Despite doing sub-group analysis and sensitivity analysis to handle the source of heterogeneity, the possible source of heterogeneity was not identified.

## Conclusion

The pooled proportions of good knowledge, positive attitude, and uptake of the HPV vaccine among adolescent schoolgirls were low in Ethiopia. The uptake of the HPV vaccine was significantly associated with good knowledge, a positive attitude, and living in an urban area. We recommend increasing adolescent schoolgirls’ knowledge, positive attitudes, and uptake of HPV vaccination through school-based seminars, health education, community mobilization, mass media, and school mini-media with an emphasis on rural areas. Furthermore, the federal Ministry of Health should design different vaccine delivery strategies for each targeted population and expand the HPV vaccination programs to include all young females**.** Future research should involve investigations of general populations' and parents' knowledge and perception of the HPV vaccine and its effect on the uptake of the HPV vaccine using a mixed-method design.

## Supplementary Information

**Additional file 1.**

**Additional file 2.**

**Additional file 3.**

**Additional file 4.**

## Data Availability

The authors confirm that the data supporting the findings of this study are available within the article [and/or] its supplementary materials. Furthermore, the corresponding author (DA) will be contacted if someone wants to request the data from this study.

## Abbreviations

ES: Effect Size
HPV: Human Papilloma Virus
OR: Odds Ratio
SNNPR: Southern Nations, Nationalities, and Peoples Region
