# Supplementary material for: Knowledge, attitude, and uptake of human papillomavirus vaccine among adolescent schoolgirls in Ethiopia: a systematic review and meta-analysis
Source: BMC Womens Health. 2023 May 20;23:279. doi: 10.1186/s12905-023-02412-1 (PMC10199506; doi:10.1186/s12905-023-02412-1)
Supplement: Supplementary file 2 — Additional file 2. [file 12905_2023_2412_MOESM2_ESM.docx]

**S2 Table:** Searching strategies for some databases to assess the pooled Knowledge, attitude, and uptake of human papillomavirus vaccine among adolescent schoolgirls in Ethiopia.

| **Databases** | **Searching terms** | **Number of studies** | **Searching period** |
| --- | --- | --- | --- |
| PubMed/ MEDLINE | ("knowledge"[MeSH Terms] OR "knowledge"[All Fields]) AND ("attitude"[MeSH Terms] OR "attitude"[All Fields]) AND ("uptake" [All Fields] OR "Practice"[All Fields] OR "utilization" [All Fields] ) AND ("papillomavirus vaccines"[MeSH Terms] OR ("papillomavirus"[All Fields] AND "vaccines"[All Fields]) OR "papillomavirus vaccines"[All Fields] OR ("human"[All Fields] AND "papilloma"[All Fields] AND "virus"[All Fields] AND "vaccine"[All Fields]) OR "human papilloma virus vaccine"[All Fields]) AND associated[All Fields] AND factors[All Fields] AND ("adolescent"[MeSH Terms] OR "adolescent"[All Fields]) AND ("schoolgirls" [All Fields] OR "females" [All Fields] OR "young adults"[All Fields]) AND ("ethiopia"[MeSH Terms] OR "ethiopia"[All Fields]) | 81 | [**From 2015/01/01 to 2022/12/23**](https://www.ncbi.nlm.nih.gov/pmc#facet_date_range_divpubdate) |
| Google scholar | "Knowledge" and "attitude" and "uptake" and "human papilloma virus" and "vaccine" and "associated factors" and "adolescent" and "schoolgirls" and "Ethiopia" | 17 |  |
| ScienceDirect | Knowledge and attitude and uptake or practice of human papilloma virus vaccine and associated factors and adolescent schoolgirls and Ethiopia | 2 |  |
| DOAJ | Knowledge and attitude and uptake human papilloma virus vaccine in Ethiopia | 2 |  |
| African Journal of Online | Knowledge and attitude and uptake human papilloma virus vaccine and associated factors and adolescent schoolgirls and Ethiopia | 3 |  |
| Gray literature (specifically unpublished studies) |  | 4 |  |
| Total searched articles |  | 109 |  |
| Finally, fulfill the eligibility criteria for our review |  | 10 |  |
